# Errors in Citation Order

**DOI:** 10.1001/jamahealthforum.2025.4768

**Published:** 2025-09-26

In the Invited Commentary titled “Understanding Model Drift and Its Impact on Health Care Policy”^1^ published online on August 15, 2025, in *JAMA Health Forum*, a number of citations to references were out of order after 2 sources by authors with similar surnames were transposed. This article was corrected online.

## References

[1] Wong A, Sussman JB. Understanding model drift and its impact on health care policy. JAMA Health Forum. Published online August 15, 2025. doi:10.1001/jamahealthforum.2025.2724 40815525

